# If you can't kill the beast, tame it: Tips for surviving WhatsApp® in medical practice

**DOI:** 10.1016/j.clinsp.2022.100156

**Published:** 2023-01-18

**Authors:** Max S. Mano

**Affiliations:** Breast Cancer Group Leader, Grupo Oncoclínicas; Faculty ‒ ESMO Breast Cancer Group; Faculty ‒ Academy of Leadership Sciences Switzerland (ALSS); Vice-chair, LACOG Breast Cancer Group, São Paulo, SP, Brazil

I have previous addressed the potential harms of unrestricted access to test reports on patients with cancer's emotional well-being and how this problem is amplified by Instant Messaging (IM) communication.^1^,^2^ Although the use of IM in patient-physician communication appears to be on the rise everywhere, in Brazil this phenomenon has reached proportions that are unknown elsewhere – with many physicians estimating that greater than 90% of their interactions with patients now occur via this app.

The pressure from patients to retire our old pages and mobile phones in favour of a texting device started to mount during 2013-2014 – flourishing years of WhatsApp® in the region; and many physicians (like me), after years of reluctance, eventually surrendered. Back in 2014, I and my team embarked on a trial period that should be followed by an assessment. The initial experience was mixed, with an observed reduction in the frequency of voice calls, which were compensated by a sharp increase in text messages – probably perceived as less intrusive by patients. However, over time, the content of the interactions tended to slide away from pure medical issues to include demands for non-urgent medical reports, appointments and every sort of non-medical issues that were previously addressed by the administrative staff; like a new chapter of the ongoing process of ‘*secretarization*’ of the medical activity – on top of the electronic health records and insurance paperwork – now reaching a new dimension with the practice of a ‘*whatsmedicine’*.

Another intriguing fact was the large number of text communication delivered during off-work hours (probably when people have the time to send them). One curious and recurrent pattern was that of messages sent at night – very commonly on Sunday – stating that ‘this is just to anticipate the tasks of the week ahead’; fortunately, at least the physician ‘doesn't need to read them right away – next morning will be fine’. Frequently, these messages come with a companion list of test reports and audios. I and my team have always perceived these unnecessary messages as particularly disturbing as they threaten a much-needed period of mental rest for the oncologist.

Another emerging synergism that deserves attention is the one between WhatsApp® and new electronic prescriptions of prescription drugs; now requests for renewals or new prescriptions pop up on the screen at any time. Some health insurers have also started requiring laboratory or imaging orders by electronic prescription only, aggravating the problem.

The recommendations from health authorities in Brazil regarding the appropriate use of social media (which includes Facebook's WhatsApp®) have been rather ambiguous – stating that ‘confidential results (i.e., almost all!) should not be sent via social media’, which physicians usually comply; however, the flow of information is often the reverse – with patients downloading all sorts of test reports (including sometimes highly confidential gene test results) and naively forwarding them via IM. Physicians are usually aware that no prescriptions or consultations can be issued via WhatsApp*®*; however, what distinguishes an actual medical act from a piece of advice, clarification or complementary information is often blurry.

Table 1 depicts the key points that characterize the practice of a ‘*whatsmedicine’*. The adoption of WhatsApp*®* to communicate with patients is a process that consolidates very quickly and is difficult to reverse. Once a physician surrenders and adjusts his practice to accommodate these demands (Table 1), this is a clear sign that he is already practising a ‘*whatsmedicine’*. I estimate that meeting 3 or more of these criteria is sufficient to establish the diagnosis.

**Table 1:** Definitions of a *whatsdoc* practicing a *whatsmedicine.*

Communicates with patients by instant messaging app >90% of the times
Concedes to do subtle ‘*whatsconsultations*’ and ‘*whatsprescriptions*’ without always requiring a formal consultation appointment
Resigns to the impossibility of deviating the messages when going on vacation and/or off work hours
Is constantly swamped with test reports that should never have been sent via social media (also because of lax confidentiality standards)
Does many small tasks that in the past were fully delegated to a clinic assistant (*secretarization*), such as:
• Helping patients to schedule appointments when the switchboard fails or is off-hours
• Taking requests of non-urgent / administrative medical reports via instant messaging
• Accepting frequent interruptions to open a patient's medical chart to verify list of workups for the next consultation appointment that the patient has lost or forgotten
• Accepting frequent interruptions to copy and paste a login/password to check an online test report of a patient
• Accepting frequent interruptions, even when off work, to generate an electronic prescription of a prescription drug

In 2020, as a reaction to this new reality, I and my team decided to adopt the WhatsApp Business® tool,^3^ which has several functions that allow the configuration of restrictions and automated responses. In our experience, this does not resolve, but attenuates the burden of these devices on practising physicians’ routines and personal lives. In Table 2, I suggest a set of practical measures that might help you survive WhatsApp*®* in medical practice.

**Table 2:** The golden rules for surviving instant messaging apps in medical practice.^a^

**Measure**	**How to accomplish this**	**Why this is important**
Employ a corporative mobile phone number with clear information about work hours	Configuration in app's ‘account settings / business hours’	Failure to do this may affect your ‘away from work’ hours and vacation periods
Configure an automatic message stating that messages sent ‘off hours’ might not be read (in case of a medical issue requiring immediate medical attention, a phone call is always required)	Configuration in app's ‘account settings / away times’	To ensure at least some rest time during off hours and limit disruptions to true medical issues
		To ensure that true urgencies/emergencies will never be missed
State that issues requiring immediate medical attention always require a phone call	Configuration in app's ‘account settings / business description’	You can easily miss a text message that could turn out to be a more serious medical situation
Make it clear that test reports should never be sent via social media	Configuration in app's ‘app account settings / business description’	In many countries this is forbidden or highly discouraged (due to lax confidentiality security in most of these apps)
Request patients to avoid audio messages	Configuration in app's ‘app account settings / business description’	Audios are highly time consuming and, in many situations, cannot be listened to
Turn off the app ‘notifications’	Configuration in ‘phone settings’	Constant notifications are highly distractive and may sometimes wake you up in the middle of the night for a non-urgent issue
Request patients to never request non-urgent reports by text message	Configuration in app's ‘app account settings / business description’	Administrative demands should only be made by email or phone call to a clinic assistant
Educate patients that text messages (especially test reports) will never replace a formal consultation appointment	Explain to your patients how to rationally use these devices in relation with their treatments	You don't want to become a ‘*whatsdoc*’, and this practice can also be dangerous to patients themselves

a Though the principles may be valid for any instant messaging app, the specific recommendations in this table apply only to WhatsApp Business®.

In a previous article,^2^ I and my co-author have briefly addressed the impact of widespread digitalization on physicians’ well-being and mental health – which haven't been in great shape for years.^4^, ^5^, ^6^, ^7^, ^8^, ^9^ With few studies published to date, we were unable to specifically evaluate the impact of WhatsApp*®* on rates of professional burnout; however, considering the scenario herein depicted, one can assume it won't be good. We are about to start a prospective study addressing both patients and physicians’ perceptions on this subject.

In summary, the use of WhatsApp*®* or similar platforms continues to spread and is gradually taking control of patient-physician communication. The author of this piece is sceptical about the effectiveness of enforced rules to regulate the practice – because the nature of the app (with a bilateral communication and no optional blockade of files/voice messages) makes this type of control unrealistic. A total ban of this type of patient-physician communication by all medical societies and health authorities is the only reasonable measure – one that will protect physicians’ well-being and mental health and patients from potential confidentiality breaches and addiction in accessing test reports to obtain an immediate – and potentially misleading – response to their diseases’ condition. In the meantime, I herein propose a set of measures that might help to attenuate the problem.

## Conflicts of interest

The authors declare no conflicts of interest.
